# Factors Influencing the Behavioural Intention to Use Cryptocurrency in Emerging Economies During the COVID-19 Pandemic: Based on Technology Acceptance Model 3, Perceived Risk, and Financial Literacy

**DOI:** 10.3389/fpsyg.2021.814087

**Published:** 2022-02-09

**Authors:** Prapatchon Jariyapan, Suchira Mattayaphutron, Syeda Noorzahrah Gillani, Owais Shafique

**Affiliations:** ^1^King Mongkut's Institute of Technology Ladkrabang Business School, King Mongkut's Institute of Technology Ladkrabang, Bangkok, Thailand; ^2^Department of Agricultural Extension and Communication, Faculty of Agriculture at Kamphaeng Saen, Kasetsart University, Nakhon Pathom, Thailand; ^3^Institute of Business, Management and Administrative Sciences, The Islamia University of Bahawalpur, Bahawalpur, Pakistan; ^4^Department of Islamic and Conventional Banking, Centre of Excellence in Inclusive and Sustainable Finance, Institute of Business, Management and Administrative Sciences, The Islamia University of Bahawalpur, Bahawalpur, Pakistan

**Keywords:** cryptocurrency, behavioural intention, technology acceptance model 3 (TAM 3), COVID 19 pandemic, subjective norm, computer self-efficacy, perceived usefulness, perceived ease to use

## Abstract

Cryptocurrency could redefine the interplay of Internet-connected world markets by eliminating constraints set by traditional local currencies and exchange rates. It has the potential to revolutionise digital markets through the use of duty-free trading. This study investigates the factors which influence the behavioural intention to use cryptocurrency based on the Technology Acceptance Model 3 (TAM 3) during the COVID-19 (SARS-COV-2) pandemic. Data were collected through a cross-sectional questionnaire from 357 Pakistani business-educated adults, including investors who had a rudimentary understanding of the technology and financial instruments. Partial least square (PLS)-based structural equation modeling (SEM) was used to test the developed theoretical framework based on the Technology acceptance model 3. The PLS model has explained 72.1% of what constitutes the behavioural intention to use cryptocurrency. Surprisingly, risk was not a major consideration. This might be due to the fact that the majority of respondents thought working with cryptocurrency was hazardous. Willingness to handle cryptocurrency risk, on the other hand, might be a stumbling block to acceptance. The most essential aspect of a cryptocurrency's success was the perceived usefulness. Moreover, the moderating role of experience was not substantiated in this study. However, perceived usefulness was identified as a partial mediator of subjective norm and the perceived ease to use. This study contributed to the literature through the application of TAM 3 (an extension of the technology acceptance models) to investigate the fundamental qualities a cryptocurrency should have in order to influence investor's behavioural intention to use it. These findings provide revolutionary insights for the present and future market players for investment planning and for improved cryptocurrencies development.

## Introduction

With a global transaction value of US$5,204 billion in 2020, the Digital Payments sector is the largest within FinTech. In 2021, the total transaction value in the Digital Payments category is expected to reach US$6.752,388 million. The overall transaction value is estimated to rise at a 12.24% annual rate (CAGR 2021–2025), culminating in a total sum of US$10,715,390 million by 2025. China has the greatest cumulative transaction value (US$2,892,494m in 2021) when compared globally (Statista Digital Market Outlook, 2021). On a daily basis, trillions of dollars are transacted on the global financial system, serving customers in billions (Tapscott and Tapscott, 2017). The annual transaction worth of Automated Clearing House system in the USA is more than forty trillion dollars, nationally representing only 20% of electronic payments (Kiviat, 2015).

In the world, the digital economy is emerging and developing rapidly, bumping all the participants in the market to bring essential variations in their activities (Mourshoudli et al., 2020). According to Patel and Shrimali (Patel and Shrimali, 2021), due to the active involvement of the middle man (mostly humans or human-operated agencies), some essential challenges, such as transparency, timeliness, traceability, security, and immutability are posed, resulting in financial loss. Virtual currency not only modernises the method of payments but can also affect the future of world currencies (Seetharaman et al., 2017).

Cryptocurrency is a sub-class of digital currency (Lee Kuo Chuen, 2015). A cryptocurrency is an essential form of digital currency because of its distinct features, unlike other digital currencies issued by organisations as centralised, monitored within a geographical region or community, or secured by fiat currency (Chuen et al., 2017). Cryptocurrency is defined by the world bank as non-fiat digital currencies which do not possess any intrinsic value, nor are secured by any primary assets, and cannot be claimed as a liability from an economic mediator (Natarajan et al., 2017).

Bitcoin as the first cryptocurrency was traded in January 2009 (Dourado and Brito, 2016). Since then, approximately 5,392 crypto-currencies are being traded with a total market capitalisation of 201 billion dollars (as of April 22, 2020). This figure is constantly rising. As of August 18, 2021, there are about 6,000 cryptocurrencies, a significant rise from just a few digital coins in 2013. Indeed, the top 20 cryptocurrencies are thought to account for roughly 90% of the overall market (Raynor de Best, 2021). Solving two long-lasting problems in computer sciences, bitcoin made cryptocurrency possible by introducing two main innovations: (1) “double-spending problem” through “peer-to-peer electronic monetary system,” and (2) “Byzantine Generals Problems” through “blockchain” (Dourado and Brito, 2016).

Satoshi Nakamoto first envisioned blockchain as a peer-to-peer digital-commodity (also known as cryptocurrency) trading system (Gupta and Sadoghi, 2021). Blockchain-based financial transactions can be distinguished into three stages: first is the initiation stage where a customer buys and sells the financial assets to access the blockchain network, second is the incorporation of stakeholders for verification of financial assets, and third is a blockchain ledger to keep records of transactions. These stages have focused on four aspects of financial transactions: the verification of assets, the transaction record maintenance, the privacy of data, and the cost of transactions (Workie and Jain, 2017).

The Federal Reserve System of the US urges improvement in the current payment system to make it secure, speedy, efficient, collaborative, and global (Federal Reserve System, 2017). Cryptocurrency has the potential of delivering these required outcomes and solving this problem at low cost with convenience (Piscini and Rosenberg, 2015). With the inauguration of bitcoin as the first cryptocurrency, world businesses and economies wanted to participate and to acquire this new financial technology. In 2010, Laszlo Hanyecz made the first transaction in bitcoin by purchasing two pizzas in exchange for 10,000 bitcoins (Bort, 2014). Nowadays, you can make payments through bitcoin in around 5,040 businesses across the world including lawyer hiring, doctor's fee claims, and car purchases (Coinmap, 2018; Usebitcoins, 2018).

The numbers of cryptocurrencies quoted in the market as purchased and sold are not definite, as nowadays, any organisation or business can generate and use its own cryptocurrency as an initial coin offering (ICO) for raising funds by using blockchain technology (Burns and Moro, 2018). The collection of ICOs are wide-ranging and its growth is increasing from year to year. In 2017, US$ 5.2 billion were collected by ICOs (Ibba et al., 2018), wherein 1,387 ICOs were issued (Fenu et al., 2018). In addition to all this, it was noticed that more than 65% of ICOs have dropped their values (Haffke and Fromberger, 2020). Therefore, the innovation and the credibility of a project proposed by a company, for which funds are raised, are the reasons behind ICOs' success (Ibba et al., 2018). The handlers' intention behind the use of cryptocurrency (bitcoin) for speculation is high as compared to financial transactions (Glaser et al., 2014). More than 50% in comparison are interested in speculative trading (Hileman and Rauchs, 2017).

By 2027, 10% of GDP will be maintained in blockchain (Shift, 2015). By 2025, the annual average growth rate will be 62.1% (Business Wire, 2017). Crypto markets have grown in the capitalisation of ~100 times which is about US$1.37 billion to US$140 billion from April 2013 to April 2020 (Coin Market Cap, 2020). As of this writing, the market value of all cryptocurrencies combined is above $2 trillion. Cryptocurrency-based loan apps and decentralised buying and selling platforms now have $65 billion in assets on their books. Over $1 billion worth of cryptocurrencies will be spent in the first quarter of 2021 (World Economic Forum, 2021). According to Coin Gecko, which analyses over 8,800 currencies, the entire market capitalisation of cryptocurrencies has already surpassed US$2.06 trillion, as the Bitcoin and other cryptocurrencies like Cardano, XRP, and Doge coin climbed on Saturday, August 14, 2021. Even during the COVID-19 Pandemic, Bitcoin climbed to US$48,152,which is its highest level since May 16, 2021 (Joanna Ossinger, 2021).

It took many years for bitcoin and other cryptocurrencies to gain popularity, but in the last few years, they have been expanding more rapidly (Madey, 2017). Cryptocurrency is becoming more widely accepted, with firms such as Microsoft, PayPal, eBay, Dell, and Expedia all accepting Bitcoin payments (Iglesias de Ussel, 2015). Experts now see blockchain technology as a viable platform for company development, particularly in high-tech, investment, and finance sectors (Vovchenko et al., 2017). Systematic Literature Review (SLR) in February 2020 anticipated that a growing number of studies on the use of cryptocurrency will be published in the near future (Lone and Naaz, 2021). Many studies have been conducted on cryptocurrencies perspectives and have been challenging cryptocurrency as a new investment opportunity (Bonneau et al., 2015; Chuen et al., 2017; Liu et al., 2019; Corbet et al., 2020; Hamakhan, 2020; Kim and Deka, 2020). However, there is still one capacity that has not been given acceptable consideration: factors affecting the use of cryptocurrency, especially in the context of Pakistan.

Nowadays, cryptocurrency generates many opportunities, such as efficient, fast, secure, collaborative, and global opportunities, and, thus, have the potential for growth in the market. Despite cryptocurrency's various opportunities, it also poses many drawbacks and problems. Firstly, Cryptocurrency has been used for money-legalising, tax dodging, illegal import transactions, extortion, and robbery of bitcoin itself (Bloomberg, 2017). This leads to the need to investigate users' knowledge about the bitcoin ecosystem with relation to confidentiality, safety, and secrecy. It was concluded that 22% of participants lost money due to self-induced faults and security gaps (Krombholz et al., 2016).

A study revealed that non-users have perceived that they are incapable of using bitcoin, have a misunderstanding about transaction privacy, and are unfamiliar with its functions (Gao et al., 2016). Secondly, a barrier in cryptocurrency development is the lack of financial and technological knowledge. In 2015, the United States conducted a study on financial knowledge and decision making measured by standard questions related to bond price, mortgage, interest rate, inflation, and risk. Respondents who were able to correctly answer all the five questions were only 14% (Lin et al., 2016). Thirdly, constraint to cryptocurrency is the risk associated with volatility in prices. A study by International Netherlands Group (ING) International Survey Mobile Banking found that, in bitcoin, 29% of European investors perceive cryptocurrencies as risky investment (Exton and Doidge, 2018). It has greater risk and price instability as compared to conventional or fiat currency. It was also revealed that cryptocurrency, especially the bitcoin blockchain, is going to illicit illegal financial transactions by using social media, big data, and enlarging bitcoin data (Turner and Irwin, 2018). Besides the volatility in cryptocurrency, like bitcoin prices, it still continues to boom on the internet. Enormous psychological thresholds on prices open due to unpredictability in cryptocurrency (Pelegrín-Borondo et al., 2015).

All this warrants investigation on the question, “what are the essential variables affecting the acceptance of cryptocurrency by the investors in emerging economies?”

In short, cryptocurrencies have potential and opportunities for growth in the emerging economies, with respect to collaborative, noticeable, effective, speedy, confidential, and safe transactions, but it also poses difficulty in using and in adaptability due to less knowledge on technological and financial activities. High volatility in prices and undefined social awareness are drawbacks of cryptocurrencies (Arias-Oliva et al., 2019). A gap in knowledge about bitcoin is due to its relatively young nature, which causes a major threat to the currency market as the value of a currency is associated with confidence in currency (Madey, 2017). Research on cryptocurrency and bitcoin greatly expands as more disciplines draw the black-chain technology to its study (Holub and Johnson, 2018). However, the innovation and complex characteristics of cryptocurrencies make its literature infrequent. TAM 3 aims to assist academics and practitioners in determining why a given technology or system may be acceptable or undesirable, along with taking appropriate actions by giving both explanation and prediction (Lai, 2017). The research was accompanied in Pakistan with a sample of university-educated adults, including investors. The motive behind selecting them is because of their financial knowledge and their elementary knowledge of computers. Moreover, business-educated adults are current and potential investors of financial instruments, most likely of cryptocurrency.

## Literature Review

### Investors' or Customers' Behavioural Intention to Use Cryptocurrency

In the technology acceptance literature, the phrase “intention to use” refers to a user's desire to use technology in the future. As the study's outcome variable has demonstrated itself to be a good indicator of actual technological use, the intention to use technology has been chosen (Ajzen, 1991; Turner et al., 2010). The majority of technology research takes place in an organisational setting, with the main goals of utilising the technology based on its effectiveness, efficiency, and usefulness. When investigating the desire to use technology in everyday life, both non-utilitarian and utilitarian motivations for usage must be reinforced with the technology viewpoint (Nysveen et al., 2005).

Technology-based research is a multidisciplinary field, including academics from the fields of media, sociology, and social psychology, which is the cornerstone of the uses and gratifications of the study (Blumler and Katz, 1974). The basic concept is that users look for pleasure in media and technology based on their unique “needs” or “motives” since research on usage and gratifications focuses on each user in daily life (Lin, 1996). Several utilitarian reasons linked to usefulness and simplicity of use have been discovered *via* investigations of uses and gratifications (Höflich and Rössler, 2001; Leung, 2001). In the case of cryptocurrencies and bitcoin, studies show that the perceived usefulness is the crucial element in the intention of whether or not to use them for electronic payments (Mendoza-Tello et al., 2018). In another cryptocurrency study, based on theory of planned behaviour (TPB), the subjective rules (social influence) and perceived behavioural control (as simple or difficult to use cryptocurrencies) are crucial (Schaupp and Festa, 2018). Individuals who see cryptocurrencies as easy to use and get favourable social influence over their use are more inclined to use them.

Bitcoin has also been investigated as a cryptocurrency. Shahzad et al. (2018) found that the perceived usefulness and perceived ease of use had a significant effect in the Chinese acceptability survey on the desire to adopt bitcoin. Multi-attribute models are frequently used to predict behavioural intention (Fishbein and Ajzen, 1977). The process through which technology becomes an integrated element of daily life is frequently described in theories. Users' perceptions of a technology's many characteristics are the subject of such models. In this literature, there are following theories that help to comprehend customers' intention to use technology.

### Technology Acceptance Theories and Literature Review

The rate at which payment systems grow, according to Hoenig (1995) and Lai (2016), is the struggle between fast technical progress and the inherent barriers to the acceptance of new products or services. There is a range of suggestions to explain why people are using and wanting to use new technologies. According to the Theory of Diffusion of Innovations (DIT) (Orr, 2003) that began in 1960, diffusion is the process through which members of a social system spread an innovation over time *via* particular routes. The idea describes the “process of distributing an innovation to members of a social system through certain channels over the period” (Rogers, 2010, p. 5).

Technology readiness (TR) refers to people's willingness to adopt and use new technology in their personal and professional lives (Parasuraman and Colby, 2001). The Theory of Task-technology Fit (TTF) (Goodhue and Thompson, 1995) is appropriate for examining actual technology usage, particularly for testing new technology and receiving feedback. In the Theory of Reasonable Action (TRA) (Fishbein and Ajzen, 1977), a person's “performance of a certain activity is determined by his or her behavioural intention (BI), which is impacted by the person's attitude and subjective norm (SN) regarding the conduct in question” (Davis et al., 1989, p. 983). The Theory of Planned Behaviour (TPB) (Ajzen, 1991) and The Decomposed Theory of Planned Behaviour (Taylor and Todd, 1995c) described the relation of beliefs to behaviour. The two initial parts are the same as the theory of reasonable action and the third aspect is the perceived control over their actions by users. Shih and Fang (2004) examined the adoption of online banking by employing the TPB and decomposed TPB.

The Technology Acceptance Model (TAM) (Davis et al., 1989), the final version of TAM (Davis and Venkatesh, 1996), is one of the most prominent and commonly employed theories, and it addressed the user's behavioural intention to use and to adopt new technology. The Technology Acceptance Model 2 (TAM2) (Venkatesh and Davis, 2000) can be classified into three broad areas. First, it worked on psychological aspects by stimulating the TAM construct (Karahanna et al., 1999). Second, it revealed the importance of the TAM construct through theoretical support (Karahanna et al., 1999). Lastly, it extended the original TAM construct by determining an addition (Karahanna and Straub, 1999; Venkatesh, 2000; Venkatesh and Davis, 2000; Koufaris, 2002).

Technology Acceptance Model (TAM) is a model established to focus on explaining how the people or users respond toward emerging technology (Hu et al., 1999). Technology Acceptance Model 3 (TAM3) (Venkatesh and Bala, 2008) combined TAM 2 (Venkatesh and Davis, 2000) and the model of determinants of perceived ease of use (PEU) (Venkatesh, 2000). In TAM 3, to explain the users' behavioural intention to use technology, a complete network of determinants is introduced. These determinants are social influence, individual differences, system characteristics, and facilitating conditions.

In the Unified Theory of Acceptance and Use of Technology (UTAUT) (Venkatesh et al., 2003), the performance expectancy, effort expectancy, social influence, and enabling circumstances are the four determinants of users' behavioural intention. In the UTAUT model, performance expectancy is made up of five comparable constructs: perceived usefulness, extrinsic incentive, job-fit, relative advantage, and result expectancies. Effort expectancy is made up of the ideas of PEU, complexity, and its extension, the Unified Theory of Acceptance and Use of Technology 2 (UTAUT2) (Venkatesh et al., 2012). This was accomplished by: (1) selecting three important dimensions from past research on both general and consumer adoption and usage of technologies; (2) altering some of the existing linkages in UTAUT's original conception; and (3) introducing new relationships consistent with the overall principles outlined by Johns (2006) and Alvesson and Kärreman (2007) on how to broaden a theory by exploiting a new context, as well as the ideas given in the TAM special issue of the Journal of the AIS (Bagozzi, 2007; Venkatesh et al., 2007) to make it more consumer friendly.

### Research Model and Hypothesis Development

In this research, TAM 3 has been used to examine the relationship between variables as TAM 3 has grown so widespread that it has been mentioned in the majority of studies on user adoption of technology (Lee et al., 2003). During the past thirty years, Technology Acceptance Model (TAM) was considered to be the most applicable and dominant theory in the community of technology (Lucas Jr and Spitler, 1999; Venkatesh and Davis, 2000) dTAM has been extensively tested with many samples in various settings and has shown to be a viable and accurate model for understanding information system acceptance and usage (Mathieson, 1991; Davis and Venkatesh, 1996). Though TAM was well-received (Yang, 2005), it concentrated on the impact of users' perceptions of the technology's utility and ease on adoption intentions (Luarn and Lin, 2005; Lai and Zainal, 2015). As a result, it is advantageous for determining innovative technologies, such as the single platform E-payment System. TAM 3 shows the relationship between subjective norm (SN) on perceived usefulness (PU), computer anxiety (CA), and computer self-efficacy (CS) on perceived ease of use (PEU). The PU, PEU, and SN have a subsequent effect on behavioural intention to use (BIU), which in turn leads to use behaviour (UB). The new relationships determined in Technology Acceptance Model 3 (TAM3) are that the experience will moderate the relationship between:

1. Perceived ease of use (PEU), and Perceived usefulness (PU),
2. Computer anxiety and Perceived ease of use (PEU), and
3. Perceived ease of use (PEU) and Behavioural intention.

Moreover, the PU has been examined as mediator between SN and BIU, as well as PEU and BIU. Also, perceived risk and financial literacy factors, other than TAM 3 factors, are used, having a direct influence on behavioural intention to use cryptocurrency. The subjective norm is described as “the extent to which a person understands that most individuals who are significant for him think that he or she must utilise the system or not” (Fishbein and Ajzen, 1975, p. 302; Venkatesh and Davis, 2000, p. 187). Perceived usefulness is described as “to the extent that a person feels that utilising an IT improves the performance of his work” (Davis et al., 1989, p. 320). Computer self-efficacy is described as “the degree to which a person feels himself or herself has the capacity to accomplish a certain task/work utilising a computer” (Compeau and Higgins, 1995, p. 121). Computer anxiety means “the degree to which someone is concerned about, or even fears when they confront the opportunity to use computers” (Venkatesh, 2000, p. 349). Perceived ease of use is defined as “The extent to which someone thinks it is effortless to use IT” (Davis et al., 1989, p. 320). Experience is defined as a person's stream of observations, interpretations of those sensations, and consequent emotions throughout a system contact (Roto et al., 2011).

Through internalisation and identification processes, the subjective norm and image will significantly influence perceived usefulness (Venkatesh and Davis, 2000). Internalisation is described as the inclusion of the belief of a referent in the structure of his own belief (Warshaw, 1980). In a variety of circumstances, including technology adoption and others, a subjective norm is present. In addition, many researchers think that this building is important (Ramayah and Razak, 2008; Lada et al., 2009; Amin et al., 2013). Ramayah and Razak (2008) studied the influence of subjective norm and intention on the acceptance of Islamic housing by postgraduate students, and reports subjective norm has influenced on behaviour intention. Lada et al. (2009) has utilised TRA reports as a subjective norm to highlight the importance of halal products as an increasingly important market power, with a direct influence on views toward the use of halal products. Subjective norm has influenced on the acceptance of Islamic household products, according to Amin et al. (2013).

In addition, research by Park (2009); Abramson et al. (2015), and Teo (2012) revealed a substantial impact of the SN on the PU and BIU. Individual differences or general attitudes about computers and computer use are reflected by Computer Self-Efficacy and Computer Anxiety (Venkatesh and Bala, 2008). Numerous empirical studies have documented the impact of self-efficacy. Examining customers' willingness to accept a diminishing partnership in house financing, self-efficacy was revealed to be one of the important elements influencing intention by Shaikh et al. (2018b). Taylor and Todd (1995a) investigated students' intentions to use a computing resource centre and found that both self-efficacy and resource-based enabling circumstances are important predictors of behavioural control. Taylor and Todd (1995b) both reported the same finding. The confidence in one's capacity to do the intended behaviour has been shown to have a major impact on the underlying motivation of users to use electronic brokerage technology (i.e., self-efficacy) (Bhattacherjee, 2000).

The more individuals utilise computers in their everyday lives, the more people are projected to experience computer issues (Beckers and Schmidt, 2001). Computer anxiety, among other issues, can represent a major obstacle to the efficient use of computers (Simsek, 2011). To some extent, computer anxiety is defined as an emotional response. It differs from negative computer attitudes, which are based on personal opinions and feelings about computers rather than emotional responses to computer use (Sam et al., 2005). Furthermore, a high degree of computer anxiety was adversely linked to the technology learning (Harrington et al., 1990), computer resistance (Torkzadeh and Angulo, 1992; Weil and Rosen, 1995), and poor job efficiency (Heinssen Jr et al., 1987). A student has more desire to learn a technology as he/she is less confident in his or her computer skills (Zhang and Espinoza, 1998).

In developing perceptions of usefulness, consumers will continue to emphasise perceived ease of use. This argument is based on action identification theory (Vallacher and Kaufman, 1996), which presents a clear distinction between the identities of high and low activity levels. Particularly, the high-level identities of individuals are related to their aims and plans, while the low-level identities are linked to ways by which their goals and plans are achieved. For instance, a high-level identity can provide a high quality report in the context of text processing software, whereas a low-level identity can be marking keys or using a particular software function (Davis and Venkatesh, 2004). While using a computer resource centre, Taylor and Todd (1995c) examined the TAM, theory of planned behaviour (TPB), and Decomposed theory of planned behaviour (DTPB) models and discovered that perceived ease-of-use had a significant influence on perceived usefulness (Taylor and Todd, 1995c). Henderson and Divett (2003); Huynh and Le Thi (2014), and Moslehpour et al. (2018) had examined the PU as a mediator and concluded that PU has a significant mediating effect between PEU and BIU and SN and BIU.

Experience, it is hypothesised, will moderate the relation between SN and PU, CA and PEU, and PEU and PU (Venkatesh and Bala, 2008). It therefore shows that the interaction with a system is “individual” and “dynamic” (Allam and Dahlan, 2013). Whereas, Dishaw and Strong (1998), under the title Experience as a Moderating Variable in a Task-Technology Fit Model, did not support the moderating effect of experience. Two beliefs impact the intention of individuals to utilise a technology, namely, perceived usefulness and perceived ease of use. The effect of external elements on behavioural purposes (e.g., design characteristics) is mediated by theoretically perceived usefulness and ease of use. In the recent two decades, there have been important empirical evidence for TAM (e.g., Adams et al., 1992; Agarwal and Karahanna, 2000; Venkatesh et al., 2003, 2007; Karahanna et al., 2006).

Perceived usefulness refers to the fact that consumers choose a service if they believe it would improve the application of technology (Ryu, 2018). In the last 10 years, a slew of empirical studies have shown that perceived usefulness has a positive impact on users' intention (Hong and Zhu, 2006; Ng and Kwok, 2017). Considering the broader perspective, if there isn't a target degree of technology adoption or service acceptability, the product or system would not work (Amoako-Gyampah, 2007). In other words, system usefulness may be considered to represent acceptance of a specific product or purpose toward a system. Many scholars have shown a strong link between perceived ease of use and attitudes toward new technology adoption in banking studies (Akturan and Tezcan, 2012; Szopiński, 2016). When consumers employ sophisticated information systems to perform financial transactions using portable mobile devices, Riquelme et al. found that perceived usefulness had a substantial impact on users' views and readiness to embrace technology. Users are more likely to accept technology services if they believe they are convenient, pleasant, and simple to use (Riquelme and Rios, 2010). The following hypothesis was formed based on the aforementioned analysis:

**H1:** SN significantly influences PU.
**H2:** SN significantly influences BIU.
**H3a:** CS significantly influences PEU.
**H3b:** CA significantly influences PEU.
**H4:** PEU significantly influences PU.
**H5:** PU mediates the relationship between SN and BIU.
**H6:** PU mediats the relationship between PEU and BIU.
**H7:** E moderates the relationship between SN and PU.
**H8:** E moderates the relationship between CA and PEU.
**H9:** E moderates the relationship between PEU and PU.
**H10:** PU significantly influences BIU.
**H11:** PEU significantly influences BIU.

The perceived risk is the consequence of a selection that reflects the difference between its end results (Gefen et al., 2003), and the potential to which it is not safe to implement innovation (Gerrard and Cunningham, 2003). The major reasons why people fear adopting new technology are risk and uncertainty (Worthington and Edwards, 2000; Pikkarainen et al., 2004). Empirical research on factors predicting the behavioural intention by Faqih (2016) defined the risk seen as an understanding of the degree of insecurity and the potential negative importance of consuming or buying a product by the consumer. Perceived risk was utilised as a factor of the behaviour of investors or consumers to use or to adopt a product (Salisbury et al., 2001; Kannungo and Jain, 2004). Moreover, perceived risk was utilised as a predictor of the use and adoption of technology (Featherman and Pavlou, 2003).

Behavioural intention has been shown to be affected by perceived risk (Kesharwani and Bisht, 2012). The studies of Chen (2008); Yang et al. (2012), and Liébana-Cabanillas et al., 2014) emphasised the necessity of considering both perceived risk and perceived trust as a significant worry that the danger of financial loss influences whether or not payment systems are adopted. Security and privacy concerns were discovered to be key factors in risk perception (Tang et al., 2003; Christou, 2006, 2007, 2011; Tan et al., 2009). One of the three most important elements in influencing cryptocurrency acceptance is perceived risks (viability, safety risk, third-party service default risk, user error risk, risk of privacy loss, counter-party fraud risk, and danger of illegal affiliation) (Nuryyev et al., 2018). More than a few modern types of research have been studied with the aim to utilise Fintech with inconsistent outcomes and have perceived risk as a factor influencing its behaviour. In their study, security certificates are considered as an important precursor to online banking (Khan et al., 2017). The perceived risk influences mobile banking in rural locations significantly in terms of descriptions (Kishore and Sequeira, 2016). The findings revealed that the security or privacy risk has a negative impact on the intention to use internet banking (Lee, 2009).

Shaikh et al. (2018a) concluded in their studies that the perceived risk has a weak direct relation with behavioural intention to use mobile banking, but it has significant importance in the preadoption process. It has influenced other factors that, in the future, have a direct effect on the behavioural intention to adopt or use. Farah et al. (2018) found in their findings that perceived risk has no influence in Pakistan's mobile banking behavioural intention. Moreover, Moon and Hwang (2018) concluded from their findings that perceived risk has no negative effect on the behavioural intention to use crowd-funding. Mendoza-Tello et al. (2018), concerning the literature on cryptocurrencies, determined that perceived risk has no importance in clarifying the intention to adopt cryptocurrencies for electronic payments. There was no evidence of a substantial and direct link between perceived risk and behavioural intention to use (Widyanto et al., 2021). Cryptocurrencies are a developing financial technology involving potential risk. Hence, the following hypothesis has been developed:

**H12:** Perceived Risk negatively influences Behavioural Intention to use.

Financial knowledge is described as the understanding of important financial concepts that allow an individual to make effective and educated financial decision-making according to their ability (Stolper and Walter, 2017). Hastings et al. (2013a) described financial literacy as the ability to make appropriate use of financial resource management knowledge and skills for lifetime financial stability. As used in academic literature, financial literacy has a range of meanings: it is used for financial products (e.g., what is a bonus vs. a stock), for knowledge about financial concepts (compounding, inflation, credit scores, and diversification), mathematical abilities or the need for numeracy essential for taking financial decisions effectively, and making financial planning. Financial knowledge as a predictive factor of financial behaviour has been studied by several researchers.

Van Rooij et al. (2011) demonstrated that financial decision-making has been influenced by financial literacy. There is a problematic issue of a lack of financial literacy if it makes people incapable of optimising their own welfare or the sort of competitive pressures required to achieve market efficiency, especially if their stake is substantial. This has evident implications for society and individual welfare (Hastings et al., 2013a). Duarte and Hastings (2012); Hastings et al. (2013b) demonstrated the large number of participants in Mexico's Social Security private account system, investing their account balance with dominant financial suppliers charging excessive charges that are not offset for greater returns and add to high management costs for the whole system. Many researches have shown broad and preventable consumer financial errors by consumers, some with insignificant financial implications. In addition to the lab experiments by Choi et al. (2010), it showed that many investors, especially those with a good level of education, fail to choose fees to reduce portfolios even when costs are the sole key characteristic of investments and dispersal of fees. Other financial errors include high interest debt holdings and lower balance sheets in the credit card (Gross and Souleles, 2002), holding taxable assets into tax-deferred accounts and non-taxable or taxable assets (Barber and Odean, 2004; Bergstresser and Poterba, 2004), borrowing from a payday lender when cheaper sources of credit are available (Agarwal et al., 2009), and paying off a mortgage quicker than the amortisation schedule allows while neglecting to contribute to a matching tax-deferred savings account (Amromin et al., 2007).

Individuals with little financial understanding are less interested in investing in equities (Lusardi and Mitchell, 2014). In the assessment of their literature entitled, “the economic importance of financial literacy,” it shows that several papers determine that the more financial knowledge a person has, the more interested in participating in and acquiring equities on financial markets they are. They mention publications from the U.S. and other nations in their research. Moreover, Stolper and Walter (2017) discussed that people's behaviour toward more saving, saving planning, financial markets participation, and intellectual selection among financial instruments are all associated with greater financial knowledge. Similarly, bad financial decision making, poorer debt management, expensive credit card practises, and additional expensive loans are all associated with lower financial knowledge.

Hastings et al. (2013b) demonstrated in their literature review that decisions taken for investments in financial instruments, utilisation of credit cards, loans on a mortgage, and saving plans for retirements are influenced by financial knowledge. Relevant findings have been described by Stolper and Walter (2017), who argue that several research papers have determined that people with high financial literacy are more thoughtful in their financial decision making. Lam and Lam (2017) examined “the relationship between financial literacy and problematic online purchasing in adults” and concluded that the development of financial knowledge in the general public especially in adults has a positive influence on the existence of problematic internet shopping. Carlin et al. studies of the elements affecting technology adoption over millennia shows that life expectancy and financial literacy have important implications for technology adoption aspirations (Carlin et al., 2017). Based on the above literature findings, financial literacy has influenced the use of financial instruments and/or products, and as known that cryptocurrency is a technological financial instrument, the following hypothesis has been developed:

**H13:** Financial Literacy positively influences Behavioural Intention to use cryptocurrencies.

**Figure 1:**
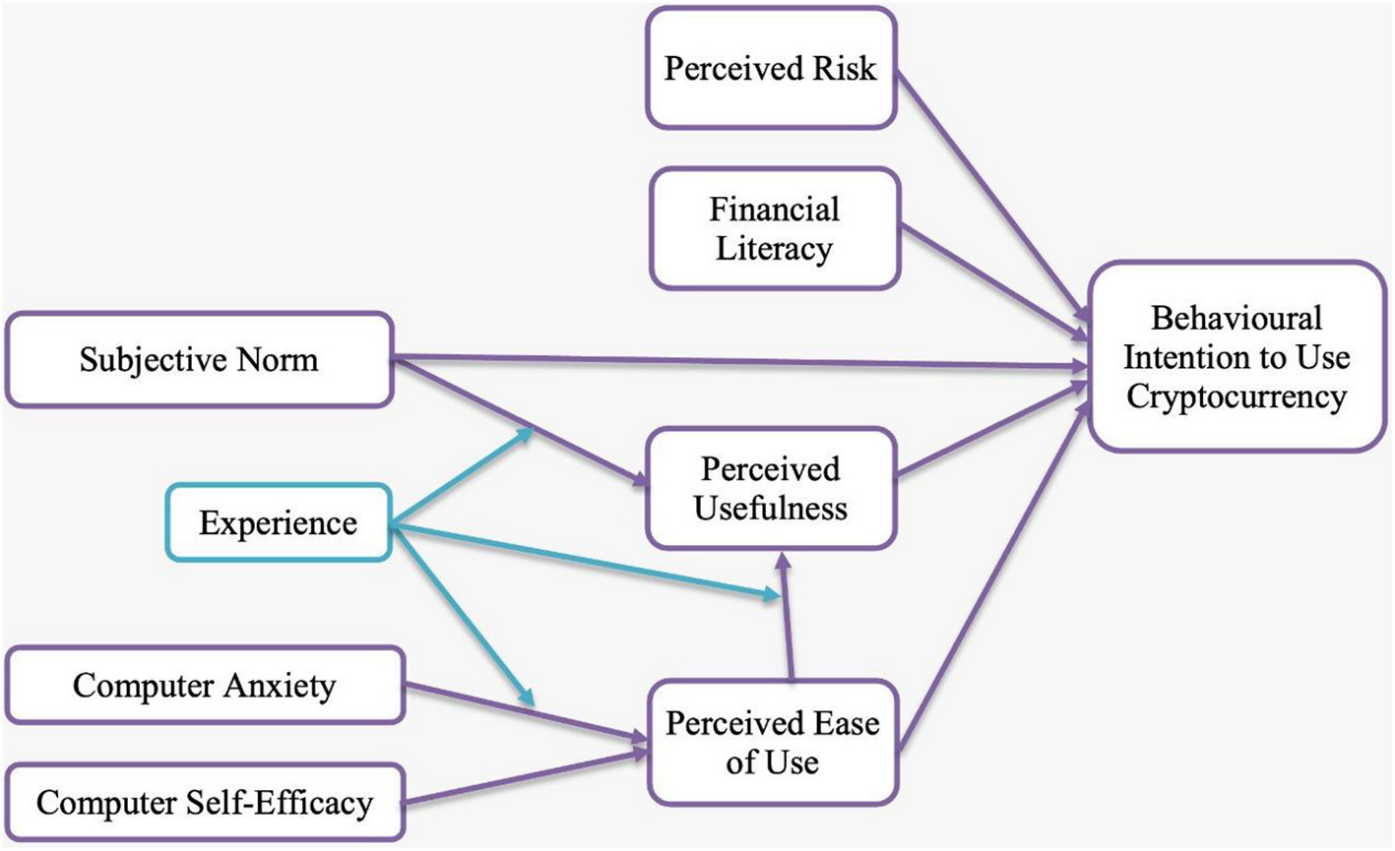
Theoretical framework.

The demonstrated relations among the examined variables are based on literature analysis of related theories. Hypotheses were developed to establish a connexion between research variables. The identified variables were used to construct a connexion, which will be translated into a theoretical framework (Figure 1) in order to acquire findings from the hypothesis required for the model test.

## Research Design and Methodology

According to Ghauri et al. (2020), the type of research determines how a study is designed. The nature of this study is quantitative to examine the relationship between variables. As in this study, the relationship between variables examined on the basis of previously proposed theories, models, and hypotheses, quantitative research is, therefore, suitable for this study (Cooper et al., 2006; Lateh et al., 2017). Similarly, Creswell and Creswell (2017) suggested that quantitative research design is the most effective means of evaluating hypotheses and is good for analysing the connexion between groups and rationalisation of interdependence among variables. In quantitative research, statistical approaches are used to collect data for hypothesis formation, testing, and for similar interpretation (Sathishkumar et al., 2013). The researcher used a cross-sectional questionnaire survey approach to collect data to investigate the topic of cryptocurrencies adoption in Pakistan's business institutions and the factors that contribute to it as suggested by other researchers (Veal, 2005; Hair et al., 2011; Myers, 2019).

### Population and Sampling Technique

With Cryptocurrencies, known as digital currency based on blockchain technology, as indicated in the introduction, it is necessary to have a minimal degree of technical and financial expertise to comprehend the way to work with it in fundamental terms. Thus, this study focused on adults, who already graduated or are studying in business schools or universities, and investors in order to gather data as they are the most engaged, informed, and valuable current or potential investors of financial markets. The cluster area sampling technique has been used for the collection of data in this study. The main reason is that the simple random sampling technique is not convenient due to the law and order situation in the COVID-19 pandemic; it has been difficult to visit all the universities of Pakistan (Rathakrishnan et al., 2021). As a result, the survey was confined to a certain location that was chosen at random. Secondly, the university-wide enrolment list of students and faculty members is not updated on the official website of Higher Education Commission of Pakistan nor is it available at the registrar office of public business schools. Thirdly, the cluster area sampling technique is used because it is the most cost-effective and time saving technique as compared with other probability sampling techniques.

### Design of Questionnaire

According to Chomeya (2010), a 7-point Likert scale is the best utilised approach for social and behavioural sciences research scaling. The Likert scale was meant to determine to what extent people agree or disagree with a given declaration (Sekaran and Bougie, 2019). The design of the questionnaire is formulated by using a 7-point Likert scale by providing more choices or options for purpose of capturing more variability in respondents' feelings and attitudes. However, a number of studies have claimed that the 7-point scale is better—one of the main reasons being that it minimises respondents' misunderstanding (Fornell, 1992; Solnet, 2006). In the current research, a 5-item scale to measure the BIU was adopted from Venkatesh and Davis (2000) and Buabeng-Andoh (2018). To measure SN, PU, and PEU, a 4-item scale adopted from the UTAUT2 by Venkatesh et al. (2012) and, recently, by Buabeng-Andoh (2018), for measuring the students' intention to adopt m-learning in Accra capital of Ghana (country in West Africa) was used. CA and CS were measured with a 4-item scale and 5-item scale, respectively (Heinssen Jr et al., 1987). A 3-item scale was used to measure the experience from Lewis and Erdinç (2017). To measure perceived risk, a 3-items scale was adopted (Arias-Oliva et al., 2019). A 3-item scale was used to measure the financial literacy in the study by Hastings et al. (2013b).

### Sample Design and Data Collection

The sampling process aimed to gather data from the selected population instead of collecting data from every demographic component (Cooper et al., 2006; Zikmund et al., 2010). The PLS-SEM analysis model is used for the analyses of data collected from the sample. Hair Jr et al. (2016) suggested that, for the SEM, minimum sample size should be about 200 respondents. Therefore, the needed sample size of the present study is minimum 200. In addition, few pieces of research on universities have been carried out by selecting business graduates, academics, and investors for the study sample. Moreover, in the context of Pakistan, response rate have been shown to be 56% by Bodla et al. (2014) and 49% by Gardner (2012). In practise, a larger sample size is preferred to prevent non-response distortion (Sekaran and Bougie, 2019). For primary data collection, an online survey link was set up and shared with the respondents via contacts/acquaintances and through social media. We requested 550 respondents for the survey, yet only 357 respondents turned in their responses (a 64.9% response rate) as presented in Table 1—which is not very uncommon as discussed in the previous chapter. Lastly, because it was an online questionnaire with set parameters, there were no missing data. The profile of the respondents is presented in Table 2.

**Table 1:** Response rate of the questionnaires.

**Response**	**Frequency/rate**
No. of questionnaires	550
Questionnaires filled	357
Questionnaire not filled	143
Response rate	64.9%

**Table 2:** Demographic profile of respondents.

**Demography**	**Description**	**No. of responses**	**Percentage**
Gender	Male Female	200 157	56.03 43.97
Age (in years)	<20 20-25 26-30 More than 30	36 183 81 57	10 51.3 22.7 16
Education	Bachelor Masters MS/MPhil PhD	205 78 45 29	57.4 21.8 12.6 8.1
Experience	None <1 1–5 6–10	150 77 105 25	42 21.6 29.4 7

## Data Analysis

The study assesses measures for convergent and discriminant validity according to Anderson and Gerbing (1988), and then constructs composite reliability using Confirmatory Factor Analysis (CFA) and SEM, in order to cheque the path links between SN, CA, CS, PU, PEU, BIU, and Experience (E) as moderator and PU as mediator. In this work, SEMs, based on a partial least square (PLS), was utilised. PLS is a well-established approach for the estimation of the path coefficients of the structural models (Ali et al., 2014). Because latent models can be constructed using small to medium samples under non-normality, the PLS approach has grown in popularity in management and marketing research over the last decade (Chin, 1998a).

### Measurement Model Results

**Figure 2:**
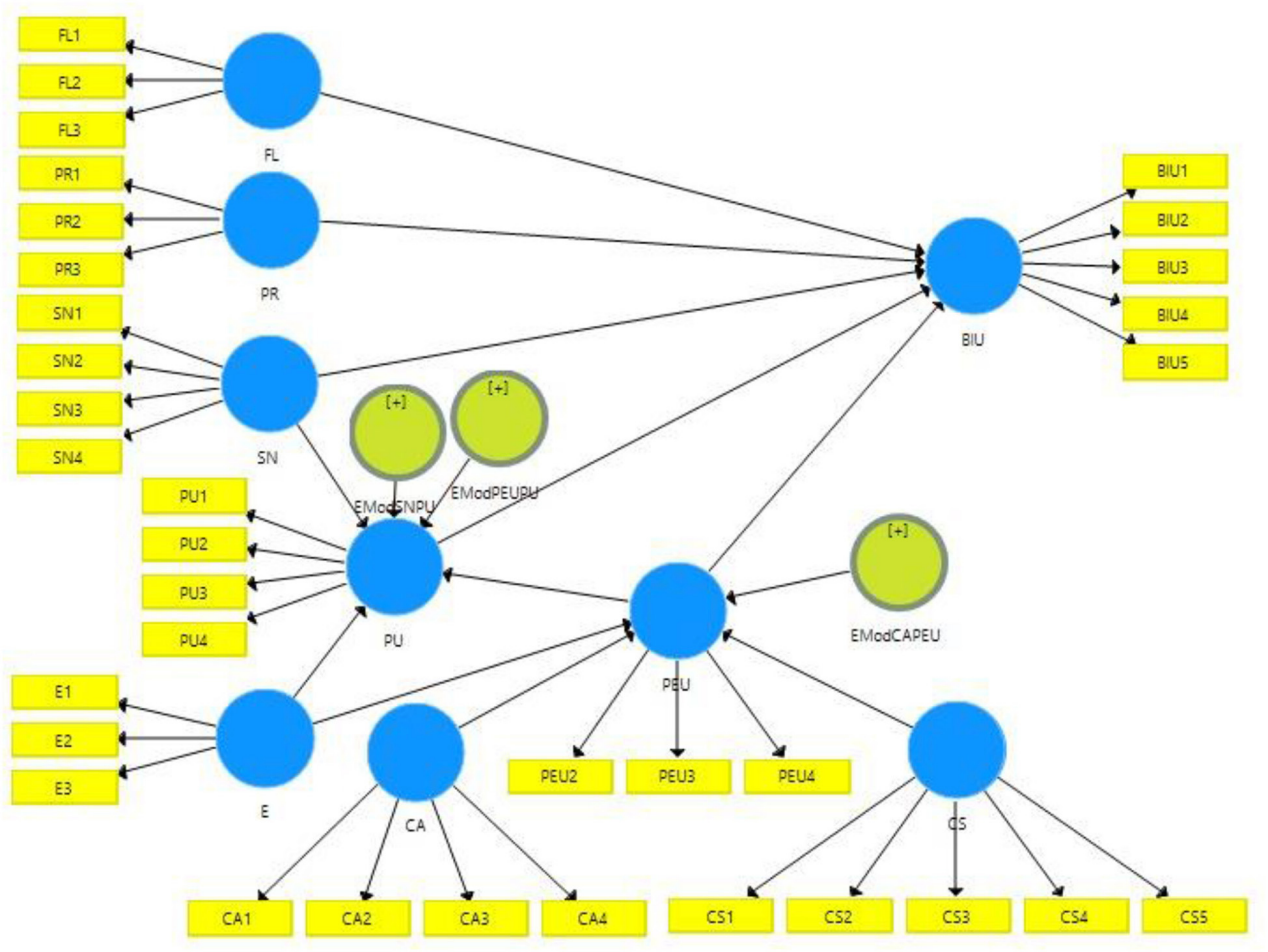
Structural model.

In this study, by using four criteria (i.e., reliability indicator of the variables/items observed, reliability of the internal consistency, discriminant validity, convergent validity, and model fit evaluation), measurement model is evaluated (Henseler et al., 2009, 2014; Sarstedt et al., 2017; Hair et al., 2019). Figure 2 represents the measurement model:

For internal consistency dependability, Hair Jr et al. (2017) suggested that Cronbach's alpha and composite reliability must be more than 0.7 and <0.95. The study revealed that all Cronbach's alpha and composite reliability values were above the threshold of 0.7, indicating that all variables have excellent internal consistency dependability. Table 3 shows the results for internal consistency reliability.

**Table 3:** Indicators loadings, composite reliability, and average variance extracted of latent variables.

**Indicators**	**Loadings**	**AVE**	**CR**	**Cronbach's Alpha**
**Behavioural intention to use**		0.789	0.949	0.933
BIU1	0.893			
BIU2	0.909			
BIU3	0.879			
BIU4	0.906			
BIU5	0.852			
**Computer anxiety**		0.654	0.901	0.869
CA1	0.612			
CA2	0.878			
CA3	0.951			
CA4	0.751			
**Computer self-efficacy**		0.727	0.930	0.906
CS1	0.854			
CS2	0.876			
CS3	0.844			
CS4	0.830			
CS5	0.859			
**Experience**		0.864	0.950	0.921
E1	0.915			
E2	0.933			
E3	0.940			
**Financial literacy**		0.762	0.906	0.844
FL1	0.893			
FL2	0.881			
FL3	0.845			
**Perceived ease of use**		0.826	0.935	0.895
PEU2	0.892			
PEU3	0.918			
PEU4	0.917			
**Perceived risk**		0.804	0.925	0.878
PR1	0.891			
PR2	0.929			
PR3	0.870			
**Perceived usefulness**		0.849	0.957	0.940
PU1	0.912			
PU2	0.921			
PU3	0.930			
PU4	0.922			
**Subjective norms**		0.807	0.944	0.920
SN1	0.897			
SN2	0.912			
SN3	0.884			
SN4	0.900			

*AVE, average variance extracted; CR, composite reliability*.

Convergent validity was established through factor loadings, Cronbach's Alpha, CR and AVE (Hair et al., 2014). The amount to which an item belonging to one variable represents the same concept is known as convergent validity (Fornell, 1994). When the value of AVE is 0.5 or more than 0.5, convergent validity is acceptable (Chin, 1998b; Hair et al., 2011, 2013). Table 3 indicates that the value of all item loadings exceed 0.6 (Chin, 1998a). CR values were higher than the suggested 0.7 (Hair et al., 2006) in addition, the value of AVE, which quantifies the degree of variance in the indicators explained for by the latent construct, for all the variables investigated in this study met or exceeded the minimum suggested cutoff value, indicating that convergent validity was achieved.

The discriminant validity of a measure refers to how effectively it is not a reflection of other factors (Ramayah et al., 2013). When a variable is observed to be distinct from other variables, it is said to have discriminant validity (Duarte and Raposo, 2010). The Fornell-Larcker and Heterotrait-Monotrate Ratio (HTMT) criteria were used to assess the discriminant validity of all variables in the current investigation. All variables' shared variance should not exceed their AVEs according to Fornell and Larcker (1981). HTML should preferably be <0.85 or 0.9. For the purpose of evaluating discriminant validity, this study used both criteria. Tables 4, 5 demonstrate that the square root of each construct's AVE (diagonal values) is greater than its associated correlation coefficients, suggesting sufficient discriminant validity (Fornell and Larcker, 1981).

**Table 4:** Discriminant validity (Fornell-Larcker criterion).

**(Fornell-Larcker criterion)**									
	**BIU**	**CA**	**CS**	**E**	**FL**	**PEU**	**PR**	**PU**	**SN**
BIU	0.888^*^								
CA	0.038	0.808							
CS	0.508	−0.140	0.853						
E	0.743	0.102	0.425	0.929					
FL	0.722	−0.003	0.495	0.671	0.873				
PEU	0.747	0.144	0.467	0.812	0.609	0.909			
PR	0.263	−0.122	0.324	0.274	0.337	0.296	0.897		
PU	0.758	0.141	0.488	0.784	0.572	0.811	0.261	0.921	
SN	0.735	0.105	0.485	0.753	0.644	0.737	0.248	0.784	0.898

* *The main diagonal displays the square root of the average variance derived from each multi-item construct*.

**Table 5:** Discriminant validity [Heterotrait-monotrait ratio (HTMT)].

**Heterotrait-monotrait ratio (HTMT)**									
	**BIU**	**CA**	**CS**	**E**	**FL**	**PEU**	**PR**	**PU**	**SN**
BIU									
CA	0.070								
CS	0.550	0.161							
E	0.800	0.101	0.461						
FL	0.813	0.046	0.566	0.759					
PEU	0.817	0.147	0.511	0.894	0.699				
PR	0.289	0.164	0.361	0.301	0.388	0.332			
PU	0.809	0.107	0.525	0.842	0.641	0.884	0.288		
SN	0.792	0.102	0.526	0.817	0.730	0.812	0.274	0.842	

Furthermore, as demonstrated in Table 6, results show that the loadings on each indicator's own construct are always larger than the cross loadings with other constructs. As a consequence of the cross-loadings criteria, the results show discriminant validity between all constructs.

**Table 6:** Cross loadings.

	**BIU**	**CA**	**CS**	**E**	**FL**	**PEU**	**PR**	**PU**	**SN**
BIU1	0.893	0.038	0.423	0.670	0.700	0.655	0.233	0.685	0.665
BIU2	0.909	0.059	0.438	0.639	0.665	0.625	0.198	0.659	0.649
BIU3	0.879	−0.019	0.472	0.657	0.618	0.664	0.289	0.656	0.679
BIU4	0.906	0.089	0.451	0.697	0.640	0.712	0.210	0.678	0.685
BIU5	0.852	−0.002	0.476	0.634	0.579	0.658	0.240	0.686	0.588
CA1	−0.059	0.612	−0.014	−0.048	0.022	−0.030	−0.163	−0.011	−0.036
CA2	−0.018	0.878	−0.158	0.057	−0.049	0.079	−0.143	0.049	0.041
CA3	0.042	0.951	−0.169	0.128	0.015	0.171	−0.067	0.144	0.131
CA4	0.036	0.751	0.004	0.046	0.003	0.082	−0.160	0.123	0.065
CS1	0.470	−0.168	0.854	0.388	0.445	0.408	0.275	0.410	0.448
CS2	0.462	−0.171	0.876	0.386	0.419	0.457	0.289	0.454	0.448
CS3	0.387	−0.099	0.844	0.311	0.357	0.331	0.257	0.395	0.334
CS4	0.394	−0.071	0.830	0.352	0.456	0.359	0.299	0.363	0.407
CS5	0.439	−0.107	0.859	0.364	0.430	0.413	0.263	0.447	0.414
E1	0.631	0.140	0.327	0.915	0.572	0.730	0.221	0.695	0.646
E2	0.712	0.095	0.439	0.933	0.642	0.748	0.257	0.745	0.718
E3	0.727	0.086	0.417	0.940	0.653	0.786	0.285	0.745	0.734
FL1	0.628	0.016	0.387	0.600	0.893	0.565	0.308	0.518	0.591
FL2	0.654	0.008	0.430	0.596	0.881	0.560	0.292	0.520	0.579
FL3	0.608	−0.042	0.482	0.560	0.845	0.466	0.282	0.458	0.516
PEU2	0.648	0.175	0.369	0.709	0.525	0.892	0.260	0.746	0.681
PEU3	0.710	0.132	0.471	0.740	0.561	0.918	0.288	0.747	0.677
PEU4	0.678	0.121	0.430	0.766	0.574	0.917	0.258	0.718	0.655
PR1	0.207	−0.119	0.252	0.179	0.232	0.238	0.891	0.224	0.166
PR2	0.258	−0.123	0.315	0.256	0.302	0.281	0.929	0.217	0.218
PR3	0.237	−0.062	0.299	0.295	0.364	0.273	0.870	0.262	0.277
PU1	0.675	0.147	0.399	0.725	0.513	0.732	0.262	0.912	0.715
PU2	0.695	0.119	0.452	0.721	0.545	0.736	0.228	0.921	0.731
PU3	0.699	0.121	0.491	0.747	0.560	0.760	0.247	0.930	0.740
PU4	0.721	0.135	0.455	0.699	0.488	0.758	0.225	0.922	0.703
SN1	0.638	0.086	0.427	0.695	0.568	0.670	0.237	0.678	0.897
SN2	0.651	0.089	0.451	0.675	0.590	0.654	0.246	0.702	0.912
SN3	0.631	0.131	0.413	0.638	0.563	0.634	0.221	0.702	0.884
SN4	0.719	0.109	0.450	0.698	0.593	0.691	0.191	0.733	0.900

Model fit (Table 7) is assessed using two valid metrics in PLS-path modelling. Henseler et al. (2014) revealed that root mean square residual (SRMR) and goodness-of-fit (GoF) are presently employed to assess how well the proposed model fits the data (Henseler and Sarstedt, 2013). The proposed range of SRMR values is 0–1. For a well-fit model, a value less than or equal to 0.05 is advised, but a value of 0.08 is also acceptable (Hu and Bentler, 1999; Hooper et al., 2008).

**Table 7:** Model fit evaluation.

	**Saturated model**	**Estimated model**
SRMR	0.048	0.052
d_ULS	1.345	1.617
d_G	0.819	0.829
Chi-Square	1696.024	1667.187
NFI	0.849	0.852

The value of the SRMR produced by PLS 3 is 0.048 structured model and 0.052 estimated model, which are considerably less than the value of 0.08. It shows that the model in question is appropriate. The normed fit index (NFI) value of almost 1 shows greater fit, according to Lohmöller (1989). The NFI is 0.849 in this research and shows that the model fits well.

Tenenhaus (2005) introduced another diagnostic tool, the goodness-of-fit index (GOF), which may similarly be used to assess model fit. According to the recommended GOF value, data corresponds perfectly with the suggested value 1 (Tenenhaus et al., 2004). For better understanding, the GOF values are split into three groups. If the figure is 0.10, the recommended model is a little amount, 0.25 is reasonable for the data, and 0.36 is a good match for the data (Wetzels et al., 2009). Table 8 shows the results of the current study's Goodness of Fit examination.

**Table 8:** Goodness-of-fit (GoF) index.

**Constructs**	**AVE**	**R square**
BIU	0.789	0.721
CA	0.654	
CS	0.727	
E	0.864	
FL	0.762	
PEU	0.826	0.687
PR	0.804	
PU	0.849	0.752
SN	0.807	
$\bar{AVE}$	0.786	
$\bar{R^{2}}$		0.72
$\bar{AVE}$ *$\bar{R^{2}}$	**0.5659**	
**GOF =** $\sqrt{\bar{{\text{R}}^{2}}\times \;\bar{\text{AVE}}}$	**0.7523**	

*We computed a goodness-of-fit value of 0.7523 for the model employed in this investigation, indicating a very excellent model fit. According to Hoffmann and Birnbrich (2012), a cut-off value of 0.36 for GoF_ Large is a suitable match*.

### Structural Model

**Figure 3:**
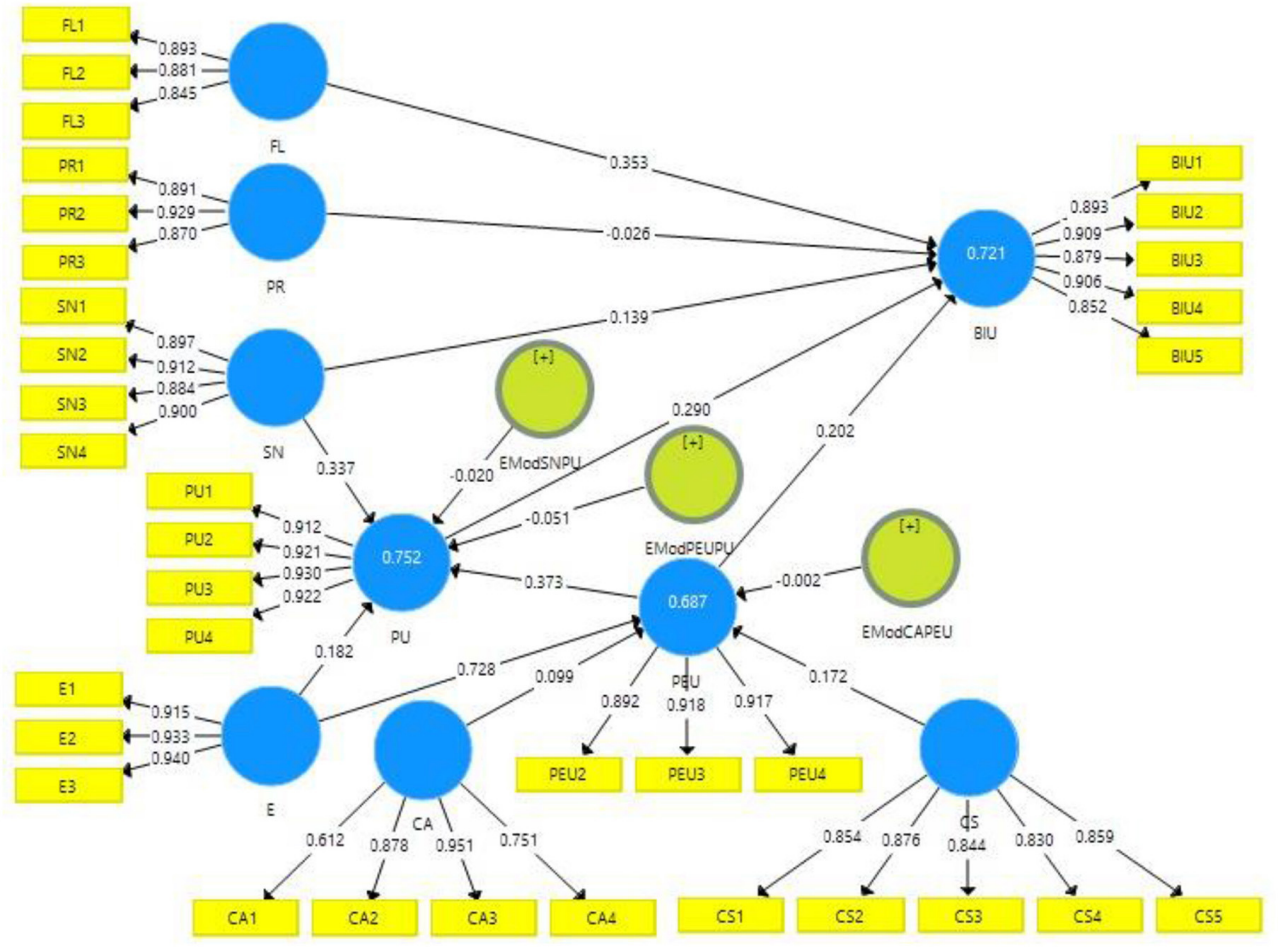
Measurement model.

The structural model evaluates significance level of path coefficient, coefficient of determination (*R*^2^), model predictive relevance (Q2), and effect size (f^2^) (Shmueli et al., 2016). The statistical significance of the weights of sub constructs and the path coefficients was evaluated using a bootstrapping approach (Chin et al., 2008). The structural model's hypothesised connexions were tested after the measurement model and goodness-of-fit were evaluated. Figure 3 represents the results of structural Model.

The explanatory strength of the model can be assessed with *R*^2^ (Wasko and Faraj, 2005). The quality and accuracy of the model are determined by the coefficient of determination (*R*^2^) (Hair et al., 2014). The extent of variance in the dependent (endogenous) variables is caused by one or more independent (exogenous) variables (Hair et al., 2006, 2014). Chin (1998b) reported that *R*^2^ is categorised into three categories: weak (0.19), moderate (0.33), and significant (0.67). Table 9 shows the results of the coefficient of determination (*R*^2^) values.

**Table 9:** Results of R^2^ values.

**Endogenous constructs**	**R square**	**R square adjusted**
BIU	0.721	0.717
PEU	0.687	0.684
PU	0.752	0.748

Perceived ease of use explained 68.6% of behavioural intention to use (*R*^2^ = 0.686). Perceived usefulness predicted 75.2% of behavioural intention to use (*R*^2^ = 0.752). Perceived usefulness and perceived ease of use, together, predicted 71.5% of behavioural intention to use (*R*^2^ = 0.715). Figure 3 also indicates the explanatory power of the predictors variable(s) on each construct is shown by the adjusted *R*^2^ values.

The f^2^ can be determined by eliminating predictor constructs from the path model, changes the *R*^2^ level, and demonstrates whether it is significantly impacting the criterion variable to remove a predictor construct (Chin, 1998b). Cohen's effect size (f^2^) is broken down into three classes: weak (0.02), moderate (0.13), and strong (0.35) (Cohen, 2013).

As mentioned in Table 10, the effect sizes for computer anxiety and computer self-efficacy are 0.027 and 0.072 on perceived ease of use viewed as weak. Subjective norm and Perceived ease to use have effect size value 0.153 and 0.151, respectively, on perceived usefulness, shown as moderate. The effect sizes for financial literacy, perceived ease of use, perceived risk, and perceived usefulness on behavioural intention to use are 0.233, 0.044, 0.002, and 0.080, respectively. Hence, following the guidelines of Cohen (2013), the effects sizes of these four exogenous latent variables on behavioural intention to use could be viewed as strong, weak, none, and moderate, respectively. According to Chin et al. (2003), even the tiniest strength of f^2^ should be addressed because it has its own distinct effect on the dependent variable.

**Table 10:** Results of effect size (f^2^).

**Endogenous constructs**	**BIU**	**PEU**	**PU**	**Effect size**
CA		0.027		Weak
CS		0.072		Weak
FL	0.233			Strong
PEU	0.044		0.151	Weak, Moderate
PR	0.002			None (No Effect)
PU	0.080			Moderate
SN	0.022		0.153	Weak, Moderate

The Geisser-Stone blindfolded test is often used for Q^2^ computation. Q^2^ illustrates how effectively gathered data may be empirically reformed using the model and the PLS parameters, based on the blindfolding technique. It also evaluates the performance of the model (Rigdon, 2014; Sarstedt et al., 2014). According to Sattler et al. (2010), this test was applied to endogenous variables with reflective measurements. A Q^2^ greater than 0 indicates that the model is predictively relevant, whereas a Q^2^ <0 indicates that the model is not predictively relevant (Fornell and Cha, 1993). Chin (1998b) split Q^2^ into three sections. The Q^2^ with a value of 0.02, represents the limited predictive relevance of the model. Whereas, the 0.15 and 0.35 structural model's Q^2^ showed medium and large predictive relevance.

As shown in Table 11, Q^2^ for perceived ease to use, perceived usefulness, and behavioural intention to use indicate acceptable predictive relevance.

**Table 11:** Results of R^2^ and Q^2^ values.

**Endogenous constructs**	**R square**	**Q square**
BIU	0.721	0.557
PEU	0.687	0.559
PU	0.752	0.625

In order to find significant path coefficients, all the hypotheses of this study were investigated by using P-statistics (under 0.05) and t-statistics (above 1.96) (Preacher and Hayes, 2004; Hair et al., 2011). The path coefficients of structural relations have been calculated and the findings are illustrated in Table 12.

**Table 12:** Structural estimates (Hypothesis testing).

**Hypothesis**	**Path relation**	**Path Cof**.	**T statistics (|O/STDEV|)**	***P***	**Decision**
H1	SN -> BIU	0.139	1.977	0.049	Supported
H2	SN -> PU	0.337	4.822	0.000	Supported
H3a	CS -> PEU	0.168	3.888	0.000	Supported
H3b	CA -> PEU	0.092	2.771	0.076	Rejected
H4	PEU -> PU	0.373	4.938	0.000	Supported
H10	PU -> BIU	0.358	4.910	0.000	Supported
H11	PEU -> BIU	0.229	3.298	0.003	Supported
H12	PR -> BIU	−0.029	0.957	0.426	Rejected
H13	FL -> BIU	0.388	6.764	0.000	Supported

The entire findings of the structural model and hypothesis testing are presented in Table 12. The findings of this study demonstrate that SN have significant influence on both BIU and PU. CS has significant influence on PEU, but CA has no influence on PEU, while PEU has significant and positive influence on PU. According to the current study finding, Perceived risk has no negative influence on behavioural intention to use. Furthermore, the data suggest that PU, PEU, and FL has significant and positive influence on behavioural intention to use.

### Mediation Analysis

Bootstrapping is one of the most rigorous and robust techniques to evaluate the mediation effect, and it is gaining traction among academics (Hayes, 2009; Zhao et al., 2010). The current study used Smart PLS 3.0 to examine the influence of PU mediating variable. Both direct and indirect outputs are examined for PU resolution. There was a substantial direct relationship between SN and BIU (*p* = 0.049) and PEU and BIU (*p* = 0.003). The relationship between SN, BIU, PEU, and BIU is still significant, but it strengthens following the addition of mediating variables such as PU (*p* = 0.003). These results demonstrated that PU partially mediate the relationship between SN, BIU, PEU, and BIU that supports our H5 and H6 hypotheses (see Tables 13, 14).

**Table 13:** SEM path coefficients of direct hypothesis.

**Hypothesis**	**Path relation**	**Path Cof**.	**T statistics (|O/STDEV|)**	***P***	**Decision**
H1	SN -> BIU	0.139	1.977	0.049	Supported
H11	PEU -> BIU	0.202	2.977	0.003	Supported

**Table 14:** Mediation assessments of perceived usefulness (PU).

**Hypothesis**	**Path relation**	**Original sample (O)**	**T statistics (|O/STDEV|)**	***P***	**VAF**
H5	SN -> PU -> BIU	0.098	2.976	0.003	0.413
H6	PEU -> PU -> BIU	0.108	2.917	0.004	0.349

### Moderation Analysis

According to Table 12, there is a strong association of SN and PEU with PU (*p* = 0.000) while CA has no association with PEU (*p* = 0.076). Hypothesis 7, 8, and 9 asserted that E moderates the relationship between SN and PU, CA and PEU, and CS and PEU. This study's findings revealed that there is no moderating role of experience on the relationship between SN and PU, CA and PEU, and PEU and PU (see Table 15).

**Table 15:** Moderation assessments of experience.

**Hypothesis**	**Path relation**	**Path Cof**.	**T statistics (|O/STDEV|)**	***P***	**Decision**
H7	EModSNPU -> PU	−0.020	0.350	0.727	Rejected
H8	EModCAPEU -> PEU	0.003	0.068	0.946	Rejected
H9	EModPEUPU -> PU	−0.051	0.837	0.403	Rejected

## Conslusion, Theoretical, and Managerial Implications

Although many studies have been conducted on cryptocurrencies perspectives and challenges (Bonneau et al., 2015), cryptocurrency is a new investment opportunity (Chuen et al., 2017), common risk factors in cryptocurrency (Liu et al., 2019), destabilising effects of cryptocurrency cyber-criminality (Corbet et al., 2020), the effect of individual factors on user behaviour (Hamakhan, 2020), advanced applications of block-chain technology (Kim and Deka, 2020), a cross-pollination of ideas about distributed ledger technological innovation through a multidisciplinary and multi-sectoral lens: insights from the blockchain technology (Lemieux et al., 2021), etc. However, literature review reveals an area that has hardly been explored: factors influencing the investors' behavioural intention to adopt or use cryptocurrency in emerging economies. This research has been conducted as a contribution to fill this gap in the literature. Moreover, an extension of the technology acceptance model (TAM3), which is considered the most appropriate model to study the behavioural intention of users toward new technology, has been used concerning cryptocurrency. The previous studies on factors affecting cryptocurrency usage have not applied to this model to examine their variables.

This objective of this study is identifying the major determinants of cryptocurrency adoption by investors, handlers, or/and customers, which will allow present and future market players to investigate the fundamental qualities a cryptocurrency should have. The rapid rise and adoption of cryptocurrencies warrants research on the factors that influence the investment behaviour regarding their adoption so that better and enticing cryptocurrencies can be developed. The study empirically contributed to a number of known linkages between variables, which were evaluated both directly and indirectly to respond to the research gap and to achieve the relevant research objectives set forth in the introductory chapter of the present study. This research has keen-sighted the relations between the variables influencing the investors' behaviour in the adoption of cryptocurrency. All the variables studied in this study are most appropriate to the structure of the cryptocurrency. This study is expected to encourage future academics to uncover other characteristics beyond those described in this study as bitcoin has a lot of potential to be studied.

### Limitations and Recommendations for Future Research

Firstly, we are concentrating on a very narrow subset of the population, namely, adults in business universities including investors, with some basic technological knowledge and Internet understanding. Despite our discussion of this choice, future research should be focused on different sectors to obtain a wider understanding of the acceptability of cryptocurrencies in society. The long-term sustainability of cryptocurrency and bitcoin mining is another concern for future studies. Intensive computational resources and significant energy consumption are necessary for the mining process, according to Krause and Tolaymat (2018), and the energy required for mining US$1 of bitcoins between 2016 and 2018 is estimated at 17 mega-joules compared to 5 mega-joules needed to mine US$1 of gold. According to this research, sustainability issues can influence the growth of cryptocurrencies. Technology and the knowledge of financial technology will continue to change shortly. In the future, a longitudinal study should, thus, track the rise of cryptocurrency acceptability and try to update the model to current conditions.

Given the current stage of technical development, both investors and customers consider running or investing in new technology assets to be highly dangerous. The perceived risk related to cryptocurrency transactions is quite high. Therefore, it should be a preliminary requirement for cryptocurrencies in the future to resolve this issue. The first “risk-free” cryptocurrencies might have a significant competitive advantage compared to the present offer. The performance of a new cryptocurrency's product and service (or current cryptocurrency innovation projects) should be prioritised as the most important element in influencing acceptance. To guarantee that potential investors perceive this value, cryptocurrency must be developed into a high-value-added service for clients, requiring significant marketing efforts. As a cryptocurrency adds value, it will be utilised more frequently. The cryptocurrency market is recommended to focus on its usefulness for investors. Moreover, all have an important effect, such as the technical know-how and technical resources required to perform cryptocurrency's operations, an investor's compatibility with technical necessities in cryptocurrency, the presence of widely agreed operating standards, as well as easy access to a support office in the event of a problem.

## Conclusion

Cryptocurrency can be viewed as a viable alternative to conventional financial services, as it can be used for both transactional and speculative purpose and is aimed to be used by a wide range of people, from aspiring entrepreneurs to investors. The decentralised architecture and peer-to-peer characteristics of the blockchain technology are highly regarded for cryptocurrency adoption.

This study concludes that the technology acceptance model factors such as subjective norm, computer anxiety, computer self-efficacy, perceived usefulness, perceived ease of use, and experience (as a moderator) influence the behavioural intention of investors and business-educated people in public universities of Pakistan to use cryptocurrency. While, in the current study, moderating role of experience between subjective norm and perceived usefulness, computer anxiety and perceived ease of use, as well as perceived ease of use and perceived usefulness has not been established. Moreover, perceived risk has influenced the behavioural intention of investors and business-educated people in public universities of Pakistan to use cryptocurrency. This study has revealed that perceived risk does not negatively influence adoption of cryptocurrency, while financial literacy has a positive and significant impact on the adoption of cryptocurrency.

However, the findings of this research revealed that investors and business-educated people residing in Pakistan have intentions to invest/use cryptocurrency while ignoring the risk associated with it. The investors considered the cryptocurrency as an opportunity and related it to their productivity and effectiveness. They perceived that using cryptocurrency is free of effort, clear, and understandable.

## Data Availability Statement

The original contributions presented in the study are included in the article/supplementary material, further inquiries can be directed to the corresponding author.

## Ethics Statement

Ethical review and approval was not required for the study on human participants in accordance with the local legislation and institutional requirements. Written informed consent for participation was not required for this study in accordance with the national legislation and the institutional requirements.

## Author Contributions

All authors listed have made a substantial, direct, and intellectual contribution to the work and approved it for publication.

## Conflict of Interest

The authors declare that the research was conducted in the absence of any commercial or financial relationships that could be construed as a potential conflict of interest.

## Publisher's Note

All claims expressed in this article are solely those of the authors and do not necessarily represent those of their affiliated organizations, or those of the publisher, the editors and the reviewers. Any product that may be evaluated in this article, or claim that may be made by its manufacturer, is not guaranteed or endorsed by the publisher.
